# An evaluation of risk-based monitoring in pragmatic trials in UK Clinical Trials Units

**DOI:** 10.1186/s13063-019-3619-6

**Published:** 2019-09-10

**Authors:** Daniel Beever, Lizzie Swaby

**Affiliations:** Clinical Trials Research Unit, School of Health and Related Research, University of Sheffield, Regent Court, 30 Regent Street, Sheffield, S1 4DA UK

**Keywords:** Risk-based monitoring, Risk assessment, Risk-adapted monitoring

## Abstract

**Background:**

Good Clinical Practice guidelines issued in 2016 encourage risk-based approaches to monitoring clinical trials. This study compared current risk assessment and monitoring approaches in UK Clinical Trials Units (CTUs) with the published guidance and makes recommendations for risk-based monitoring in pragmatic trials.

**Methods:**

An online survey of UK Clinical Research Collaboration registered CTUs was administered via email invitation. Forty-nine units were invited, and 23 responded. Respondents were also invited to share copies of risk assessment templates.

**Results:**

Most CTUs reported using remote combined with on-site monitoring. All reported undertaking a risk assessment for Clinical Trials of Investigational Medicinal Products (CTIMPs) and 21 units did so for non-CTIMPs. Most CTIMP risk assessments used MHRA (Medicines and Healthcare products Regulatory Agency) classifications, although some also employed staff judgement. Almost all units based their monitoring on perceived risk level; this number was higher for CTIMPs (*n* = 22) than for non-CTIMPs (*n* = 19). In most cases, monitoring plans were produced. More CTUs revisited risk assessments during trials in CTIMPs (*n* = 21) than in non-CTIMPs (*n* = 18). Small numbers of units reviewed the monitoring approach always (*n* = 4) or sometimes (*n* = 9) and few used the reflection to guide future monitoring.

**Conclusions:**

A high proportion of UK CTUs are using risk-based monitoring in the UK, as recommended by guidelines, for both CTIMPs and non-CTIMPs. This has the potential to make trials more efficient and reduce costs. However, there appears to be a lack of reflection on the value of these revised approaches. There may be a benefit in CTUs collaborating nationally to improve processes for reflection and making changes during the life course of a trial.

**Electronic supplementary material:**

The online version of this article (10.1186/s13063-019-3619-6) contains supplementary material, which is available to authorized users.

## Background

Monitoring of clinical trials forms one of the main approaches to ensuring that quality standards are delivered in line with International Council for Harmonisation of Technical Requirements for Pharmaceuticals for Human Use Good Clinical Practice (ICH-GCP) guidelines [1]. Trial sponsors are expected to have monitoring arrangements in place to ensure that regulatory obligations are met, the safety and well-being of participants are maintained, and scientific integrity is retained [1]. The ICH-GCP guidance does not specify methods to be used but recommends “considerations such as the objective, purpose, design, complexity, blinding, size and endpoints of the trial” [1].

Historically, trial monitoring has been onerous, involving numerous on-site visits and up to 100% source data verification (SDV) [2] and having large implications for trial management and budgets. SDV is the process by which the data collected for a trial are compared with the original source of information. More recently, there has been a shift toward increased remote monitoring conducted by the trial sponsor or coordinating trials unit or both [3]. Central checks can be carried out on electronic records, consent forms and overall performance of participating sites [3], highlighted by the recent development of metrics in this area [4]. Although this may reduce the number or duration of visits, central monitoring has its own limitations, including access to the source data and reliance on sites maintaining data collection records. A 2012 survey highlighted that of 48 UK Clinical Research Collaboration (UKCRC) registered Clinical Trials Units (CTUs), 35% indicated that all trials had a documented monitoring plan, and most CTUs used some level of central monitoring, in some cases combined with on-site monitoring [5].

In 2016, the integrated addendum to ICH-GCP was published, building on the guidance jointly published by the Medicines and Healthcare products Regulatory Agency (MHRA), the Department of Health, and the Medical Research Council in 2011 [6], encouraging sponsors to develop more systematic and risk-based approaches to monitoring [7]. This uses the initial risk assessment completed early on in a trial to assess the level of monitoring required. For example, if a trial is assessed as high-risk, more frequent site visits may be required, whereas low-risk trials may not require any on-site monitoring [3]. Risk-based approaches may include focusing SDV on specific critical data points [8]. ICH-GCP recommendations state that “statistically controlled sampling may be an acceptable method for selecting the data to be verified”, suggesting that 100% SDV is not always necessary [1]. Other risk-based approaches may include reducing activities required on-site, such as obtaining participant consent to allow documents (e.g., consent forms) to be sent to the coordinating centre for central review [8], and resolving data queries remotely through interrogation of electronic systems. In 2012, 53% of CTUs surveyed used a risk assessment to determine the level of monitoring required [5].

Although guidelines now recommend a risk-based monitoring approach, it is not known how widely this process has been adopted across UK CTUs. A recent study in Ireland, for example, identified that only 21% of respondents had performed risk-based monitoring [9]. Furthermore, the very nature of a risk-adaptive approach suggests that this should be revisited throughout a trial, assessing whether the chosen monitoring approach remains adequate following trial protocol changes and whether new risks have been identified. There is currently limited evidence regarding reflections on chosen monitoring approaches at the end of a trial. This would provide insight into lessons learnt and inform decisions about future trials.

We therefore developed a survey, administered to representatives from UKCRC registered CTUs, to determine current monitoring practices and how these approaches are being reflected upon at the end of a trial. Responses were considered in relation to existing literature and best practice guidelines on risk-based monitoring to allow us to make recommendations for undertaking, and reflecting upon, risk-based monitoring in pragmatic trials.

## Methods

The survey was developed by using Google Forms and structured, where possible, using skip logic so that respondents could avoid having to scroll through irrelevant questions. The content was developed taking into account existing literature on the topic, particularly focusing on reflection and learning from approaches used, and through discussion with colleagues in Sheffield CTU.

Almost all of the questions used tick boxes to minimise the burden on respondents. The researchers undertook extensive testing of the survey prior to its launch, particularly in relation to the use of the skip logic.

The survey had five sections: general information about the trial portfolios and approaches to monitoring, the initial risk assessment of trials, monitoring approaches and adapting to risks identified in trials, reflecting on the monitoring approach taken and the use of this information, and a “catch-all” for any further information that participants wished to provide. The full survey is reproduced in Additional file 1.

Questions were asked separately for Clinical Trial of an Investigational Medicinal Product (CTIMP) and non-CTIMP studies. A CTIMP is a trial looking at the safety or efficacy of a medicine or placebo, as defined by the Medicines for Human Use (Clinical Trials) Regulations (2004). Non-CTIMP studies do not involve investigational medicinal products and do not fall within the scope of the Medicines for Human Use (Clinical Trials) Regulations (2004).

The survey was designed to be completed on a unit-level basis (i.e., one response per unit), and CTU directors were identified as the ideal recipients; they were provided with the option of identifying other staff if they felt they were not the best person to complete the survey. Contact was made at an early stage with the UKCRC Network to discuss dissemination of the survey and it was agreed that it would be distributed via their CTU director email list, which had the contact details of all UK CTU directors (*n* = 49).

A link to the survey was contained within the email sent out, which also had a participant information sheet attached. As the survey was designed to be anonymous, consent was considered to be implied by the completion of the survey. Also for this reason, the survey could not be designed to restrict multiple responses from the same unit or person. Therefore, the documentation provided specified that there should be only one response per unit.

Follow-up reminder emails were sent out by the Network at 2 and 4 weeks after the original invitation. These contained the same information, along with a copy of the survey. The design of the survey meant that questions were presented over a number of separate pages and so suggestions were received that it would be helpful for potential respondents to see the whole survey.

Descriptive statistics, through the use of count data, were used to present data on quantitative information gathered from completed responses. Narrative summary was used to present information from the answers to qualitative questions and supplementary information provided by respondents.

## Results

### Overview of current practices within Clinical Trials Units for risk assessments and monitoring

The response rate of the survey was 47% (23/49 CTUs), and all respondents indicated that their units coordinated both CTIMP and non-CTIMPs; the vast majority (*n* = 18) coordinated more than 10 non-CTIMPs and a significant number of those (*n* = 6) coordinated more than 30. Numbers of CTIMPs were smaller, and most units (*n* = 14) coordinated fewer than 10. Further details are provided in Table 1.

**Table 1:** How many current trials does your unit coordinate? (*n* = 23)

Number of units coordinating each type of trial		
	CTIMPs	Non-CTIMPs
Fewer than 5	7	3
Between 5 and 10	7	2
More than 10	9	18*

*Six units coordinate more than 30 trials. *Abbreviation*: *CTIMP* Clinical Trial of an Investigational Medicinal Product

More than half of the respondents reported that their risk assessment processes did not differ between CTIMPs and non-CTIMPs (*n* = 13), and the other 10 respondents stated that there were differences between the types of project.

Of the 23 responses, 12 indicated that the CTU would make the decision on the level of monitoring required, in terms of the proportion of on-site versus remote monitoring. Four said that the sponsor would make this decision, and five suggested that both the sponsor and the CTU would decide on the level of monitoring. A further two respondents selected “other”: one stated that decisions were made by the CTU with input from the chief investigator, and the other stated that it would be the sponsor making the decision for CTIMPs and the CTU deciding for non-CTIMPs.

There was a clear split in the approach to risk assessment based on trial type; nearly half (*n* = 10) of the respondents indicated that the risk assessment process differed depending on whether the trial was a CTIMP or not. The most popular approach to monitoring for both CTIMP (*n* = 10) and non-CTIMPs (*n* = 13) was the use of mainly remote monitoring, with some on-site monitoring, although the use of remote monitoring only was rare (*n* = 2) and occurred only in the case of non-CTIMPs (Table 2).

**Table 2:** What type of monitoring does your Clinical Trials Unit use? (*n* = 23)

Number of units coordinating each type of trial		
	CTIMPs	Non-CTIMPs
Only on-site monitoring	1	1
Mostly on-site monitoring with some remote	5	2
On-site and remote monitoring in equal proportion	7	5
Mostly remote monitoring with some on-site	10	13
Only remote monitoring	0	2

*Abbreviations*: *CTIMP* Clinical Trial of an Investigational Medicinal Product

Almost all undertook a risk assessment for at least some of their trials regardless of whether they were CTIMPs (*n* = 23) or not (*n* = 21). See Table 3 for further information.

**Table 3:** Do you undertake a risk assessment for your trials? (*n* = 23)

Number of units coordinating each type of trial		
	CTIMPs	Non-CTIMPs
Yes	23	21
All	20	14
Most	3	3
Some	0	3
Unknown frequency	0	1
No	0	2

*Abbreviation*: *CTIMP* Clinical Trial of an Investigational Medicinal Product

### Assessment and categorisation of risk

Of the 23 CTUs responding, 19 stated that their risk assessment for a CTIMP would use the MHRA categorisations (A/B/C) whereas the remaining four stated that this would not be the case. The MHRA categorises study risk by using the definitions below:

- Type A: no higher than the risk of standard medical care
- Type B: somewhat higher than the risk of standard medical care
- Type C: markedly higher than the risk of standard medical care.

Tables 4 and 5 provide more information on how risks are assessed.

**Table 4:** Does the risk assessment assess and categorise each individual risk or the risk of the trial as a whole? (*n* = 23 for CTIMPs, *n* = 19 for non-CTIMPs)

Number of units coordinating each type of trial		
	CTIMPs	Non-CTIMPs
Individual risks	15	12
Whole study	4	3
Both	4	4

*Abbreviation*: *CTIMP* Clinical Trial of an Investigational Medicinal Product

**Table 5:** How is a risk assessment undertaken? (*n* = 23 for CTIMPs, *n* = 20 for non-CTIMPs)

Number of units coordinating each type of trial		
	CTIMPs	Non-CTIMPs
Using numerical scores	3	2
Using staff judgement	6	6
Using both	13	11
Unknown	1	1

*Abbreviation*: *CTIMP* Clinical Trial of an Investigational Medicinal Product

### Management of risk

For the question “Does the risk assessment tool indicate specific monitoring approaches to be used to mitigate the risks identified for a study?” (Table 6), there were 22 responses relating to CTIMPs and 19 responses for non-CTIMPs. For CTIMPs, 13 indicated that the risk assessment tool does indicate specific monitoring approaches to be used, but the picture was more uncertain for non-CTIMPs and only nine respondents stated that this was the case. In one response, for each type of trial, this information was unknown. This was because this question was not answered, although previous answers suggested that a risk assessment was undertaken for both types of trial.

**Table 6:** Does the risk assessment tool indicate specific monitoring approaches to be used to mitigate the risks identified? (*n* = 22 for CTIMPs, *n* = 19 for non-CTIMPs)

	CTIMPs	Non-CTIMPs
Yes	13	9
No	9	10

*Abbreviation*: *CTIMP* Clinical Trial of an Investigational Medicinal Product

Despite this, almost all based their monitoring approach on the perceived level of risk for the trial for both CTIMPs (*n* = 22/23) and non-CTIMPs (*n* = 19/21). See Table 7 for further information.

**Table 7:** Is the type of monitoring used dependent on the level of risk of the study? (*n* = 23 for CTIMPs, *n* = 21 for non-CTIMPs)

	CTIMPs	Non-CTIMPs
Yes	22	19
No	1	2

*Abbreviation*: *CTIMP* Clinical Trial of an Investigational Medicinal Product

### Monitoring plans

For those respondents who indicated that their risk assessment and monitoring processes differed between CTIMPs and non-CTIMPs (*n* = 10), nine reported producing a monitoring plan for all CTIMPs and the other respondent reported that this was the case for most CTIMPs. Only five respondents reported producing a monitoring plan for all non-CTIMPs; one reported “most” and one reported “some”. Three respondents noted that monitoring plans were not produced at all for non-CTIMPs.

Where respondents reported that their risk assessment and monitoring processes were the same for all trials (*n* = 13), a monitoring plan was produced in nine units for all trials and four units for most trials.

### Response to changes in risk assessment

Twenty-one out of twenty-two respondents reported that risk assessments were revisited throughout the course of a CTIMP and 18 out of 23 for non-CTIMPs (Table 8). In one case, it could not be determined whether a risk assessment was revisited during a non-CTIMP, as the respondent previously reported that risk assessments were completed for non-CTIMPs but did not provide an answer to this question.

**Table 8:** How often is the risk assessment revisited? *(Participants may have provided multiple answers; n = 21 Clinical Trials Units)*

	CTIMPs	Non-CTIMPs
At a fixed time point (e.g., yearly)	9	5
Routinely after each protocol amendment	10	10
In reaction to a specific issue/event	12	13
Not applicable	2	4

*Abbreviation*: *CTIMP* Clinical Trial of an Investigational Medicinal Product

Almost all respondents stated that reassessments were used to adapt the initially agreed monitoring approaches for CTIMPs (*n* = 20). This was slightly fewer but still a majority for non-CTIMPs (*n* = 16). One response for non-CTIMPs could not be determined, as an earlier answer suggested that monitoring plans were revisited for non-CTIMPs but the answer provided to this question contradicts that.

### Reflections

Of the 23 respondents, four reported that the monitoring approach is always reflected upon at the end of the trial, nine reported that the approach is sometimes reflected upon, and 10 reported that it is not reflected upon at all (Table 9).

**Table 9:** Are reflections on monitoring approaches used, documented? (*n* = 13)

		Are these reflections documented anywhere?	
		Yes	No
Is the monitoring approach reflected upon at the end of the trial?	Yes	1	3
	Sometimes	3	6

For those who reported documenting reflections at the end of a trial, respondents reported documenting these as follows. In one case, it was not recorded where this information is documented.

- “Minutes of the Quality Management Group that reviews and approves Risk Assessment and Monitoring Plans”
- “Central files in the Quality Assurance department - review of non-compliances/issues/additional monitoring required”
- “We have a lessons learnt database where we encourage both positive and negative lessons to be documented and the review team would suggest potential actions to either disseminate or act upon to improve for ongoing/future studies”.

The use of reflections to inform future trials is detailed in Table 10. For those who reported that this information is used to guide monitoring approaches for future trials, additional information was provided as follows:

- “It may influence how we cost for increased (or decreased) frequency, intensity, triggers or type of monitoring”.
- “Helped us understand what is manageable and effective”.
- “Previous learning is always taken into account”.
- “Our monitoring strategy is generally based around resource and key areas such as informed consent and eligibility. Information from previous trials will be used to improve methods and outcomes of monitoring although the overall strategy largely remains unchanged”.
- “Informal intelligence will guide future monitoring in similar studies. Individual trial teams are also likely to take forward lessons learned to their next trials”.
- “If actions are required to be performed to improve processes, this is fed into the QMS [Quality Management System]”.
- “Lessons learned from each study guide planning for the next”.
- “Shared learning between staff. Staff involved in original project invited to discuss monitoring approach for next study”.

**Table 10:** Are reflections used to guide monitoring approaches in future trials?

		Is the information used to guide monitoring approaches for future trials?		
		Yes	No	Not applicable
Is the monitoring approach reflected upon at the end of the trial?	Yes	2	2	0
	Sometimes	7	0	2
	No	2	0	8

Respondents then were asked how they attempted to ensure consistency in the assessment of risk across their trials. Eighteen responded to this question and some provided multiple answers.

- Standard templates are used for all risk assessments (*n* = 7).
- Quality Assurance Manager/team has involvement in/oversight of completed risk assessments (*n* = 5).
- Same core staff/review committee review or have input into risk assessments or both (*n* = 4)
- Sponsor process (*n* = 2)
- Input of senior staff with guidance in standard operating procedures (*n* = 1)
- Completed by allocated research-and-development staff member (*n* = 1)
- Input of senior staff (*n* = 1).

## Discussion

Nearly half of the registered UK CTUs responded to this survey, many with significant trial portfolios, thereby providing a good overview of the current approach to risk-based monitoring in the UK. Much of the information received was not surprising; more non-CTIMPs were undertaken than CTIMPs, and the approach to monitoring and risk assessments in CTIMPs indicated an understandably greater level of scrutiny and caution than in non-CTIMPs.

It is interesting to note that when compared with the results of the work of Tudur Smith in 2012 [5], a significantly larger percentage of trials units currently have a monitoring plan for all trials (61%, *n* = 14/23; compared with 35%). Furthermore, whilst the percentage of trials across units using risk assessment to guide the monitoring approach has remained largely unchanged (53% to 51%, *n* = 21/41 responses; Table 6), almost all use a risk-adaptive approach to monitoring (93%, *n* = 41/44 responses; Table 7).

The use of a risk-adaptive approach is clearly important in terms of ensuring that monitoring is cost-effective and appropriate to the needs of the trial. It is encouraging to see that almost all units are revisiting their risk assessments during their trials, albeit most often in a reactive manner to a specific issue or event, and that almost all are also using the information gathered to adapt initially agreed monitoring approaches.

However, it is unclear from this survey how significant such changes are in terms of the approach taken to monitoring. Whilst the survey does indicate that monitoring is almost entirely based on the assessed risk level of the trial, there is understandably going to be a significant use of staff judgement in making such an assessment. Such judgement, without clear mechanisms for sharing information amongst colleagues about how decisions are made, creates the potential for inconsistency in monitoring approaches within units.

The risk assessment templates and associated documentation provided by some of the respondents (*n* = 4) appeared to be thorough in their design, capturing a variety of information, such as mitigating factors, processes to be followed (including for risk assessment review), and both pre- and post-award factors. However, of these examples provided, only one of the four units indicated that they always reflect on their monitoring approach at the end of the trial, meaning that a lot of valuable information is captured and then not used.

In terms of reflections on monitoring approaches more widely, this was much more mixed across the units, and almost half did not reflect at the end of a trial at all. Even where reflection did take place, this was often not documented. This raises questions as to how consistency is maintained as well as how and whether learning takes place. In cases where units did reflect on their approach to monitoring, the responses provided indicated that this provides valuable learning, particularly in terms of making improvements for future trials (in terms of trial costing and eligibility and consent, for example).

### Strengths and limitations

Working with the UKCRC Network on recruitment to this study facilitated access to CTUs via a contact with whom they were already familiar and ensured that all registered trials units were given the opportunity to participate. The recruitment figures were consistent with the previous survey work carried out in this area [5]. A particular strength of this study has been the ability to draw comparisons with previous work in this area through the use of similar questions. Whilst only around half of registered trials units participated in the survey, this study was able to gather valuable information in an area of limited prior research. The study was also reported in accordance with guidelines for survey research [10] Additional file 2.

The limited response to the survey is an obvious limitation of this work (though a common feature in research more generally), which makes it difficult to assess how generalisable our results are in relation to the state of monitoring across the UK more widely. In addition, whilst monitoring reflections were a particular element on which this study sought information, the focus of questions was only on post-trial reflections; it would have been useful to know what, if any, reflection takes place during the course of a trial too.

Furthermore, there were issues of consistency in some responses; one respondent provided an answer that was contradicted by a later answer, and another unit completed the survey twice, providing slightly different responses. Attempts were made to clarify these issues but this was possible in only one such case, as providing contact details was optional. It is not known why one unit completed the survey twice; where responses differed, these were excluded from the results.

There was also an issue related to viewing the survey form as indicated by one potential respondent asking for a copy of the survey prior to commencing completion. This allowed the respondent to review the questions before answering, seeking input from colleagues where required, prior to submitting their response. Although a PDF copy of the survey questions was included with the reminder email, partial responses could not be saved using the survey tool. This may have made it more difficult for respondents completing the survey prior to the reminder being sent.

The design of the survey, which was largely option-based and had limited amounts of free text, also meant that assumptions sometimes had to be made from the information provided. This was perhaps not ideal but this was a conscious decision by the research team to ensure that the survey was not burdensome to complete and that we received as many responses as possible. Where assumptions were made, these were a consensus between the research team and were fully documented.

An assumption was also made around interpretation of the word “pragmatic”; the authors considered this to refer to trials which have real-world application, not necessarily just late-phase randomised controlled trials. Therefore, it was expected that the study would cover the vast majority of trials conducted within the UKCRC CTU Network, but admittedly respondents may have interpreted the term differently. Specifically, the definition of “pragmatic” is perhaps most widely considered to apply to late-phase (phase III and IV) trials [11].

Finally, a potential limitation could be non-response bias. We do not know whether the CTUs that did respond are representative of all UKCRC CTUs. Whilst the target population are all UKCRC-registered, implying a certain level of consistency, the figures in Table 1 suggest that those who did respond have relatively large trial portfolios. This could mean that monitoring practices are more established in a larger unit than perhaps smaller or more recently registered units. Further work could try to determine how representative a sample our respondents were in comparison with the UKCRC CTUs as a whole.

### Implications for practice

This research demonstrates that whilst the use of monitoring continues to grow, there is some variation in how such monitoring is delivered. It is suggested that the findings from this research should be used as a starting point for discussion at the UKCRC Network level, perhaps around the need for greater collaboration and resource-sharing on this topic.

### Future research

As indicated previously, there are obvious limitations to this research in providing only a “snapshot” of the current approach to risk-based monitoring in UK. What our research has found suggests that there would be value in a more detailed, qualitative exploration of the topic.

Greater standardisation of processes, including reflecting on monitoring approaches and recording this information, is likely to have benefits beyond the units themselves in terms of providing greater confidence to research sponsors and investigators around the processes that trials units have in place in this area.

## Conclusions

This study provides a useful update on the use of risk-based monitoring, indicating the increased use of a risk-adaptive approach in the UK. Responses demonstrated that most units have established approaches in place around monitoring, including the use of risk assessments.

Despite this, our survey indicates that there is little reflection on the monitoring approach taken at the end of the trial, documented or otherwise. Those units that did undertake such reflection were able to demonstrate through their responses the value that this provides in developing staff knowledge and improving processes for the benefit of future trials.

## Additional files

Additional file 1: Risk Based Monitoring Survey. (PDF 87 kb)

Additional file 2: EQUATOR Network Survey Reporting Checklist. (DOCX 14 kb)

## Data Availability

The datasets generated and analysed during the present study are not publicly available as the authors do not have permission to share these data. Analysis was undertaken through the use of descriptive statistics, and all data generated are presented in the article either as individual comments or as aggregate-level data.

## Abbreviations

CTIMP: Clinical Trial of an Investigational Medicinal Product
CTU: Clinical Trials Unit
ICH-GCP: International Council for Harmonisation of Technical Requirements for Pharmaceuticals for Human Use Good Clinical Practice
MHRA: Medicines and Healthcare products Regulatory Agency
SDV: Source data verification
UKCRC: UK Clinical Research Collaboration
