# Supplementary material for: An evaluation of risk-based monitoring in pragmatic trials in UK Clinical Trials Units
Source: Trials. 2019 Sep 10;20:556. doi: 10.1186/s13063-019-3619-6 (PMC6734501; doi:10.1186/s13063-019-3619-6)
Supplement: Supplementary file 1 — Risk Based Monitoring Survey. (PDF 87 kb) [file 13063_2019_3619_MOESM1_ESM.pdf]

# An Evaluation of Risk Based Monitoring in Pragmatic Trials

You are being invited to participate in a survey to evaluate current risk based monitoring practices used in pragmatic trials, within UKCRC Registered Clinical Trials Units. We have provided information below, including how the study will be conducted, to help you decide whether or not you wish to take part. Please take time to read the information and ask the study team if anything is unclear. Participation in this survey is entirely voluntary.

For further information, please see the information sheet provided to you in the invitation email and available via this link: https://drive.google.com/file/d/0B_gT_r2NbSmvUUJQRmdEU3ZHMEE/view

Please note that any information you enter will be stored and processed using services provided by Google. These services have been the subject of careful assessment to ensure they comply with UK data protection law and the University's own privacy policies.

## Section 1: General information

1. **Does your CTU coordinate both CTIMPs and non-CTIMPs?**
   *Mark only one oval.*

   - CTIMPs only
   - Non-CTIMPs only *Skip to question 3.*
   - Both *Skip to question 4.*

## Section 1: General information

2. **How many current CTIMP studies does your unit coordinate?**
   *Mark only one oval.*

   - <5
   - 5-10
   - >10

*Skip to question 7.*

## Section 1: General information

3. **How many current non-CTIMP studies does your unit coordinate?**
   *Mark only one oval.*

   - <5
   - 5-10
   - 11-30
   - >30

*Skip to question 7.*

## Section 1: General information

4. **How many current CTIMP studies does your unit coordinate?**
   *Mark only one oval.*

   - <5
   - 5-10
   - >10

5. **How many current non-CTIMP studies does your unit coordinate?**
   *Mark only one oval.*

   - <5
   - 5-10
   - 11-30
   - >30

6. **Does the risk assessment and monitoring process differ between the two?**
   *Mark only one oval.*

   - Yes *Skip to question 9.*
   - No

## Section 1: General information

7. **Which of the following does your CTU use?**
   *Mark only one oval.*

   - Only on-site monitoring
   - Mostly on-site monitoring with some remote
   - On-site and remote monitoring in equal proportion
   - Mostly remote monitoring with some on-site
   - Only remote monitoring

8. **Who makes the decision on the level of monitoring required? (I.e. in terms of content, rather than approval of processes / documentation)**
   *Mark only one oval.*

   - Sponsor
   - CTU
   - Other: ____________________

*Skip to question 12.*

## Section 1: General information

9. **Which of the following does your CTU use for CTIMPs?**
*Mark only one oval.*

- Only on-site monitoring
- Mostly on-site monitoring with some remote
- On-site and remote monitoring in equal proportion
- Mostly remote monitoring with some on-site
- Only remote monitoring

10. **Non-CTIMPs?**
*Mark only one oval.*

- Only on-site monitoring
- Mostly on-site monitoring with some remote
- On-site and remote monitoring in equal proportion
- Mostly remote monitoring with some on-site
- Only remote monitoring

11. **Who makes the decision on the level of monitoring required? (I.e. in terms of content, rather than approval of processes / documentation)**
*Mark only one oval.*

- Sponsor *Skip to question 13.*
- CTU *Skip to question 13.*
- Other: ______________________ *Skip to question 13.*

## Section 2: Initial risk assessment

12. **Do you undertake a risk assessment for your studies?**
*Mark only one oval.*

- Yes - All *Skip to question 20.*
- Yes - Most *Skip to question 20.*
- Yes - Some *Skip to question 20.*
- No *Skip to question 31.*

## Section 2: Initial risk assessment

13. **Do you undertake a risk assessment for your CTIMP studies?**
*Mark only one oval.*

- Yes - All
- Yes - Most
- Yes - Some
- No

14. **Non-CTIMP studies?**
*Mark only one oval.*
- ◯ Yes - All
- ◯ Yes - Most
- ◯ Yes - Some
- ◯ No

## Section 2: Initial risk assessment

If you do not undertake risk assessments for either CTIMPs, non-CTIMPs or both, please leave the relevant questions blank.

15. **Does the risk assessment for the CTIMP study use the MHRA categorisations (A/B/C)?**
*Mark only one oval.*
- ◯ Yes
- ◯ No

16. **Does the risk assessment assess and categorise each individual risk, or the risk of the CTIMP study as a whole?**
*Tick all that apply.*
- ☐ Individual risks
- ☐ Whole study

17. **Non-CTIMP?**
*Tick all that apply.*
- ☐ Individual risks
- ☐ Whole study

18. **How is such assessment undertaken for a CTIMP? (If other, please specify)**
*Mark only one oval.*
- ◯ Using numerical scores
- ◯ Using staff judgement
- ◯ Using both
- ◯ Other: ______________________

19. **Non-CTIMP?**
*Mark only one oval.*
- ◯ Using numerical scores
- ◯ Using staff judgement
- ◯ Using both
- ◯ Other: ______________________

*Skip to question 23.*

## Section 2: Initial risk assessment

20. **Does the risk assessment for the CTIMP study use the MHRA categorisations (A/B/C)?**
*Mark only one oval.*

- Yes
- No
- N/A (our unit does not coordinate CTIMPs)

21. **Does the risk assessment assess and categorise each individual risk, or the risk of the study as a whole?**
*Tick all that apply.*

- [ ] Individual risks
- [ ] Whole study

22. **How is such assessment undertaken? (If other, please specify)**
*Mark only one oval.*

- Using numerical scores
- Using staff judgement
- Using both
- Other: ____________________

*Skip to question 27.*

## Section 2: Initial risk assessment

23. **Does the risk assessment tool indicate specific monitoring approaches to be used to mitigate the risks identified for a CTIMP study?**
*Mark only one oval.*

- Yes
- No

24. **Non-CTIMP?**
*Mark only one oval.*

- Yes
- No

25. **Is the type of monitoring used dependent on the level of risk of the CTIMP study?**
*Mark only one oval.*

- Yes
- No

26. **Non-CTIMP?**
*Mark only one oval.*

- Yes
- No

*Skip to question 29.*

# Section 2: Initial risk assessment

27. **Does the risk assessment tool indicate specific monitoring approaches to be used to mitigate the risks identified?**

*Mark only one oval.*

- Yes
- No

28. **Is the type of monitoring used dependent on the level of risk of the study?**

*Mark only one oval.*

- Yes
- No

*Skip to question 31.*

# Section 3: Monitoring approaches and adapting to identified risks

29. **Do you produce a monitoring plan for your CTIMP studies?**

*Mark only one oval.*

- Yes - All
- Yes - Most
- Yes - Some
- No

30. **Non-CTIMPs?**

*Mark only one oval.*

- Yes - All *Skip to question 32.*
- Yes - Most *Skip to question 32.*
- Yes - Some *Skip to question 32.*
- No *Skip to question 32.*

*Skip to question 32.*

# Section 3: Monitoring approaches and adapting to identified risks

31. **Do you produce a monitoring plan for your studies?**

*Mark only one oval.*

- Yes - All *Skip to question 34.*
- Yes - Most *Skip to question 34.*
- Yes - Some *Skip to question 34.*
- No *Skip to question 34.*

# Section 3: Monitoring approaches and adapting to identified

## risks

32. **Is the risk assessment revisited throughout the course of the CTIMP study?**
*Mark only one oval.*

- Yes
- No
- N/A (no risk assessment is undertaken)

33. **Non-CTIMP study?**
*Mark only one oval.*

- Yes *Skip to question 35.*
- No *Skip to question 35.*
- N/A (no risk assessment is undertaken) *Skip to question 35.*

*Skip to question 35.*

## Section 3: Monitoring approaches and adapting to identified risks

34. **Is the risk assessment revisited throughout the course of the study?**
*Mark only one oval.*

- Yes *Skip to question 39.*
- No *Skip to question 41.*
- N/A (no risk assessment is undertaken) *Skip to question 41.*

## Section 3: Monitoring approaches and adapting to identified risks

35. **How often is the risk assessment revisited for a CTIMP?**
*Tick all that apply.*

- [ ] At a fixed timepoint (e.g. yearly)
- [ ] Routinely after each protocol amendment
- [ ] In reaction to a specific issue / event
- [ ] N/A

36. **Non-CTIMP?**
*Tick all that apply.*

- [ ] At a fixed timepoint (e.g. yearly)
- [ ] Routinely after each protocol amendment
- [ ] In reaction to a specific issue / event
- [ ] N/A

37. **Is this reassessment used to adapt the initially agreed monitoring approaches for a CTIMP?**

*Mark only one oval.*

- Yes
- No
- N/A

38. **Non-CTIMP?**

*Mark only one oval.*

- Yes
- No
- N/A

*Skip to question 41.*

# Section 3: Monitoring approaches and adapting to identified risks

39. **How often is the risk assessment revisited?**

*Tick all that apply.*

- At a fixed timepoint (e.g. yearly)
- Routinely after each protocol amendment
- In reaction to a specific issue / event

40. **Is this reassessment used to adapt the initially agreed monitoring approaches?**

*Mark only one oval.*

- Yes
- No

# Section 4: Reflections / maintaining standards

41. **Is the monitoring approach reflected upon at the end of the study? (For example, was the chosen approach to monitoring sufficient / suitable?)**

*Mark only one oval.*

- Yes
- Sometimes
- No

42. **Are these reflections documented anywhere? (For example, in a local learning log, or the final monograph)**

*Mark only one oval.*

- Yes
- No
- N/A

43. **If yes, please give details of where these are documented and how (for example, through statistics for non-compliances, or staff opinion pieces). If no, please give details of what form these reflections take:**

44. **Is the information used to guide monitoring approaches for future studies?**
*Mark only one oval.*

- Yes
- No
- N/A

45. **If yes, please give details:**

46. **How do you attempt to ensure consistency in the assessment of risk across your studies?**

## Section 5: Further information

If you are happy to share a copy of your risk assessment tool template, then please send it to d.a.beever@sheffield.ac.uk or e.a.swaby@sheffield.ac.uk. We will use these to capture information on the approaches being taken, such as the types of risks being assessed.

If you choose to share your risk assessment then we will know who you and your unit are, but this information will not be shared outside of the research team. In addition, we will only be able to link survey responses with risk assessment templates provided if you provide your details below.

47. **If you wish to provide any further information in relation to the questions asked or risk based monitoring at your CTU more generally, please do so here:**

48. **If you decide to share your risk assessment separately and are happy for this to be linked to your survey response, please provide your name and / or the name of your unit:**

49. **If you are happy to be contacted for further information related to your survey response / risk assessment template provided, then please provide your email address below:**

Powered by
Google Forms
