# Supplementary material for: An evaluation of risk-based monitoring in pragmatic trials in UK Clinical Trials Units
Source: Trials. 2019 Sep 10;20:556. doi: 10.1186/s13063-019-3619-6 (PMC6734501; doi:10.1186/s13063-019-3619-6)
Supplement: Supplementary file 2 — EQUATOR Network Survey Reporting Checklist. (DOCX 14 kb) [file 13063_2019_3619_MOESM2_ESM.docx]

| **Checklist Item** | **Reported on Page Number** |
| --- | --- |
| 1) Explain the purpose or aim of the research, with the explicit identification of the research question | Page 3-4 |
| 2) Explain why the research was necessary and place the study in context, drawing upon previous work in relevant fields | Page 3-4 |
| 3a) State the chosen research method and justify why this was chosen | Page 4 |
| 3b) Describe the research tool | Page 4 |
| 3c) Describe how the sample was selected and how data were collected | Page 4 |
| 4) Describe and justify the methods and tests used for data analysis | Page 5 |
| 5) Present the results of the research | Page 5-10 |
| 6) Interpret and discuss the findings | Page 10-12 |
| 7) Present conclusions and recommendations | Page 12 |

**Good practice in the conduct and reporting of survey research – Kelley et al, 2003**
